# CBT-E informed practice in an adult outpatient service: How effective is it in a real world setting?

**DOI:** 10.1186/2050-2974-3-S1-O2

**Published:** 2015-11-23

**Authors:** Lara Ryan, Miles Bore, Stella Dyer, Dyani Nevile

**Affiliations:** 1 University of Newcastle, Callaghan, NSW, Australia; 2 Centre for Psychotherapy, Newcastle, NSW, Australia

## 

Eating disorders are one of the most dangerous psychiatric illnesses, yet amongst the most complex to treat. Remission rates remain poor and are complicated by non-treatment factors. Recently, a shift has been made to enhanced Cognitive Behavioural Therapy (CBT-E) that is transdiagnostic in its scope. This project aimed to examine the efficacy of the current treatments offered for patients with eating disorders at the Centre for Psychotherapy. 'CBT-E-informed' was one of these treatments.

## Method

Participants were recruited from the Centre for Psychotherapy's treatment program for eating disorders. The use of CBT-e has been defined as "informed" because the therapists who were trained in CBT-e were trialling it for the first time, and no adherence measures were undertaken. Treatment outcome measures were administered at baseline, after six months of treatment, after 12 months of treatment, and again at one year post treatment follow up. It was part of a larger study that originally aimed to examine all treatment modalities offered. As such, 17 participants were offered CBT-e, three were offered ACT, and two were offered conversational model therapy. The treatments were not compared due to small numbers.

## Results

Participants identified as having been seen using ‘CBT-E informed’, significantly improved on measures of eating disorder psychopathology, both globally and at subscale level, as measured by scores on the Eating Disorder Examination Questionnaire. Participants also demonstrated improvements in work and social adjustment.

## Conclusion

From the broader data set, ‘CBT-E informed’ was able to demonstrate significant treatment gains and further our knowledge on factors affecting treatment implementation. Several measures were found to predict those who dropped out of treatment, giving insight into the non-treatment factors that complicate recovery.

